# Correlation of serum IGF-1, AGEs and their receptors with the risk of colorectal cancer in patients with type 2 diabetes mellitus

**DOI:** 10.3389/fonc.2023.1125745

**Published:** 2023-02-20

**Authors:** Zeng Chen, Qiao Hong

**Affiliations:** The Second Affiliated Hospital of Harbin Medical University, Harbin, China

**Keywords:** type 2 diabetes, colorectal cancer, IGF-1, IGF-1R, AGEs, RAGE, sRAGE

## Abstract

**Background:**

According to epidemiological evidence, people with type 2 diabetes mellitus have a higher risk of developing colorectal cancer.

**Objective:**

To examine the relationship between colorectal cancer (CRC) and serum levels of IGF-1, IGF-1R, AGEs,RAGE and sRAGE in patients with type 2 diabetes.

**Methods:**

By using RNA−Seq data of CRC patients from The Cancer Genome Atlas (TCGA) database, we divided the patients into normal group(58 patients)and tumor group(446 patients), and analyzed the expression and prognostic value analysis of IGF-1,IGF1R and RAGE. Cox regression and the Kaplan-Meier method were used to determine the predictive value of target gene on clinical outcomes in CRC patients. In order to further combine CRC with diabetes research,one hundred forty-eight patients hospitalized in the Second Hospital of Harbin Medical University from July 2021 to July 2022 were enrolled and divided into CA and control groups. There were 106 patients in the CA group, including 75 patients with CRC and 31 patients with CRC+T2DM; the control group comprised 42 patients with T2DM. Circulating levels of IGF-1, IGF-1R, AGEs, RAGE, and sRAGE in the serum of the patients were measured using Enzyme-Linked Immunosorbnent Assay (ELISA) kits, and other clinical parameters were also measured during hospitalization. Statistical methods used were χ² test, independent samples t-test and Pearson correlation analysis were. Finally, we controlled for confounding factors and used logistic multi-factor regression analysis.

**Results:**

Bioinformatics analysis showed that IGF-1, IGF1R and RAGE were highly expressed in CRC patients, and the patients with high expression also showed significantly lower overall survival rate. Through Cox regression analysis, IGF-1 can be used as an independent influencing factor of CRC. In the ELISA experiment, serum AGE, RAGE, IGF-1, and IGF-1R levels were higher in the CRC and CRC+T2DM groups than in the T2DM group, but the serum sRAGE concentrations in these groups were lower than those in the T2DM group (P < 0.05). Serum AGE, RAGE, sRAGE, IGF1, and IGF1R levels were higher in the CRC+T2DM group than in the CRC group (P < 0.05). In CRC+T2DM patients, serum AGEs were correlated with age (p = 0.027), and the serum AGE levels in these groups were positively correlated with RAGE and IGF-1 levels (p < 0.001) and negatively correlated with sRAGE and IGF-1R levels (p < 0.001). After correcting for confounding factors based on logistic multiple regression analysis, the effects of age, serum IGF-1 and IGF-1R on the development of CRC in patients with T2DM were statistically significant (p<0.05).

**Conclusion:**

Serum IGF-1 and IGF-1R levels independently influenced the development of CRC in patients with T2DM. Furthermore, IGF-1 and IGF-1R were correlated with AGEs in CRC patients who also had T2DM, suggesting that AGEs may influence the development of CRC in T2DM patients. These findings suggest that we may be able to lower the risk of CRC in the clinic by regulating AGEs through the regulation of blood glucose levels, which will affect IGF-1 and its receptors.

## Introduction

A severe threat to human health is posed by CRC, which is currently the second most frequent malignancy in terms of mortality after lung cancer (1). In 2020, it was predicted that there would be more than 1.9 million new cases of CRC, 935,000 deaths, and an increase in CRC incidence in China. T2DM has placed a significant burden on patients and society globally because of an increase in morbidity and death in recent years; it has been predicted that 700 million people will have T2DM by 2045 (2). Findings from epidemiological studies have suggested that T2DM is a risk factor for CRC, with approximately 20-40% of T2DM patients at risk for CRC and a worse prognosis for these patients (3). The underlying pathophysiological mechanisms of T2DM and CRC include hyperglycaemia, insulin-IGF-1 axis signalling, hyperinsulinaemia, inflammation caused by adipose tissue dysfunction, gastrointestinal motility disorders, and immune damage. These risk factors are similar to those for T2DM and CRC, which include obesity, low levels of physical activity, and a high-calorie, high-fat diet.

Advanced glycation end products (AGEs) include exogenous and endogenous. The former represents absorption from food or generated inside an organism (4). Exogenous AGEs include Glycolaldehyde-derived(Glycol-AGEs), Glyceraldehyde-derived(Glycer-AGEs), Methylglyoxal-derived(MGO-AGEs),Glyoxal-derived(GO-AGEs),3-Deoxyglucosone-derived(3-DG-AGEs). Methylglyoxa(MGO) is believed to contribute significantly to intracellular AGEs formation, not only due to its higher reactivity but also for its multiple origin *via* glycolysis (5). Among them, MGO is produced by T2DM patients in a hyperglycaemic state and can modify amino acids in proteins by reacting with arginine, cysteine and lysine residues to form AGEs (6). The main mechanism by which AGEs exert their biological functions is through binding to receptor for advanced glycation end products (RAGE), which acts as a fuel for CRC development, triggering the activation of a series of adverse cellular effects that promote oxidative stress, inflammation and tumorigenesis (7, 8). RAGE is a transmembrane multiligand receptor encoded by advanced glycation end-product-specific receptor(AGER), which can be recruited into exosomes and exists in serum and plasma, and is highly expressed in exosomes under the influence of tumors. So it can use the standard techniques of exosome analysis, such as ELISA, protein blot and mass spectrometry (9, 10). RAGE signalling can be blocked by targeted lysogenic cleavage of the RAGE ectodomain (cleaved RAGE: cRAGE), producing a soluble form (sRAGE) that is released from cells and appears in the circulation. sRAGE is a decoy receptor for AGEs that prevents inflammatory responses; circulating sRAGE is not only a biomarker for diabetes and complications but also may be a predictive marker for the development of malignant tumours (11). According to the literature (12), RAGE genes and sRAGE alterations play regulatory roles in the development of many malignancies. Recent clinical studies have found that prediagnostic sRAGE concentrations are negatively associated with colorectal cancer risk in men, which is possibly associated with SNPs within the ADAM10 gene and RAGE shedding (13).

Chronic hyperinsulinaemia in T2DM patients leads to elevated levels of insulin-like growth factor (IGF), which includes IGF-1 and IGF-2, a class of peptides structurally similar to insulin, and its related receptors, such as IGF-1R and IGF-2R. Among them, IGF-1 serves as an indicator that can predict early T2DM and affect its complications. igf-1 may be correlated with AGEs in CRC development. IGF-1 activates proinflammatory signalling *via* mitogen-activated protein kinase (MAPK) (by binding to IGF-1R) and the phosphatidylinositol 3-kinase (PI3K)/serine-threonine kinase (AKT) pathway, both of which affect colorectal epithelial proliferation, differentiation, and apoptosis and contribute to the development of CRC (14). In contrast, the activation of the PI3K/Akt pathway by AGEs requires the transactivation of IGF-1 receptors, a mechanism involving RAGE (15). RAGE, an immunoglobulin-like superfamily receptor, can also transactivate IGF-1 receptors (15).

Therefore, we hypothesized that serum AGEs influence the development of CRC in T2DM through IGF-1 and its receptors. The aim of this study was to examine the correlation between circulating levels of serum IGF-1, IGF-1R, AGEs, RAGE, and sRAGE and the risk of developing CRC in patients with T2DM.

## Materials and methods

### Bioinformatics analysis

A dataset of patients with CRC containing gene expression profiles and paired clinical information was downloaded from the publicly available TCGA database (https://portal.gdc.cancer.gov), including normal group(58 patients)and tumor group(446 patients). Subsequently, differences in gene expression of IGF-1, IGF-1R, and RAGE between groups and the effect of the gene on patient survival were evaluated. Finally, independent risk factors were analyzed using COX regression. All analyses were performed in the R environment, and plots were constructed using the ggbeeswarm and ggplot2 package.

### Patients

We recruited patients with colorectal cancer who were hospitalized in the Department of General Surgery of the Second Affiliated Hospital of Harbin Medical University from July 2021 to July 2022. The study was approved by the Medical Ethics Committee of the Second Affiliated Hospital of Harbin Medical University, and informed consent was obtained from all participants. The participants included in this study included patients with colorectal cancer combined with type 2 diabetes, patients with colorectal cancer alone, patients with diabetes alone. Patients with diabetes mellitus were diagnosed with type 2 diabetes mellitus according to the 1999 World Health Organization criteria and diagnostic criteria of the Chinese guidelines for the prevention and treatment of type 2 diabetes mellitus. The diagnosis of colorectal cancer was confirmed by histopathological diagnosis. We divided the participants into CA and control groups. Patients with type 2 diabetes combined with colorectal cancer (T2DM+CRC) and patients with colorectal cancer alone (CRC) were categorized as the CA group, and patients with type 2 diabetes alone were categorized as the control group.

Inclusion criteria (1): CRC patients were initially operated on in our hospital and diagnosed according to histopathological analysis (2); CRC patients had not received radiotherapy or other anticancer treatments before surgery; and (3) T2DM patients met the 1999 World Health Organization criteria and the diagnostic criteria of the Chinese guidelines for the prevention and treatment of type 2 diabetes mellitus (16).

Exclusion criteria (1): patients with comorbid type 1 diabetes (2); patients with other combined malignant tumours; and (3) patients with inflammatory bowel disease, severe cardiovascular disease, chronic renal insufficiency, or severe liver dysfunction.

### Clinical data

Sex, age, height, weight, BMI, fasting blood glucose level, maximum tumour diameter, degree of tumour differentiation, lymph node metastasis, serum CEA level, IGF1 concentration, IGF1R concentration, AGE concentration, RAGE concentration, and sRAGE concentration were among the clinical data we collected. Body weight in kilograms divided by height in square metres (kg/m2) yielded the body mass index (BMI). Participants were defined as obese if their body mass index was greater than or equal to 25 kg/m2. The degree of differentiation was based on pathological findings for TNM staging. Blood glucose levels were measured using an automated analyser (Hitachi 7600, Tokyo, Japan).

### Laboratory analyses

For all participants, serum was harvested from blood samples obtained while participants were in a fasted state at the time of diagnosis. Serum samples for the analysis of IGF-1, IGF-1R, AGE, RAGE and sRAGE were collected by the immunology laboratory and stored at -80°C prior to analysis. Enzyme-linked immunosorbent assay (ELISA) kits (AGEs: Shanghai jingkang,JLC6718; RAGE: Shanghai jingkang,JLC 6087;Srage: Shanghai jingkang,JLC5525;IGF-1: Shanghai jingkang,JLC 7140;IGF-1R: Shanghai jingkang,JLC7141) stored in a refrigerated environment were removed and left at room temperature for 30 minutes prior to experimental use according to the instructions, with three measurements taken as an average.

### Statistical methods

The data conforming to a normal distribution are expressed as the mean ± standard deviation. The χ² test was used for intergroup comparisons of count data. Between-group comparisons of patients’ serum levels were performed using the independent-sample t test (for equal variances) and Welch’s t test (for unequal variances). Pearson correlation analysis was used to compare the correlation between two serum levels. Finally, logistic multifactor regression analysis was used. The test was a two-sided test, and P<0.05 was considered statistically significant. All statistical analyses were performed using SPSS software (IBM Corp. Released 2019. IBM SPSS Statistics for Windows, version 26.0. Armonk, NY: IBM Corp).

## Results

### Expression of IGF-1,IGF1R and RAGE in CRC and Survival outcomes analysis

**Figure 1:**
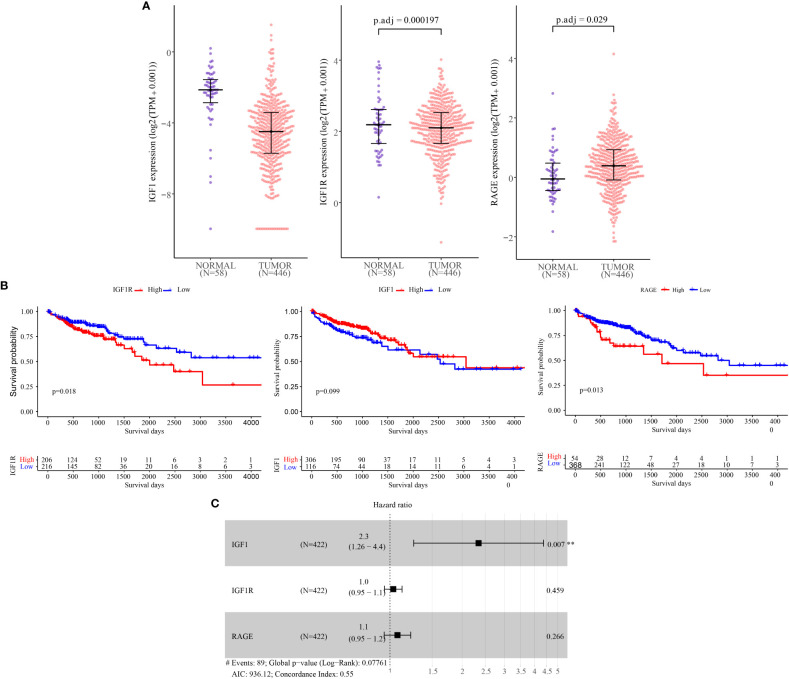
IGF-1, IGF-1R and RAGE mRNA levels and prognostic value analysis in CRC tissues according to RNA−Seq data from TCGA Data Bank. **(A)** Expression of IGF-1, IGF-1R and RAGE in normal colon tissues (n = 58) and according to tumor stage of CRC (n = 446). **(B)** Kaplan-Meier plots of overall survival **(C)** Independent risk factor analysis. ** Represents “p ≤0.01”.

To evaluate the expression of IGF-1,IGF1R and RAGE in CRC and normal person, and to explore its correlation with clinical prognosis, we initially analyzed the data in the TCGA database. We found that the expressions of IGF-1, IGF1R and RAGE in colorectal cancer tissues were different from those in normal tissues (**Figure 1A**).

In our further survival analysis of patients with TCGA colorectal cancer demonstrated that tumor expression of IGF-1R and RAGE was associated with shorter overall survival (Figure, P=0.018;Figure,P=0.013) while IGF-1 was not correlated with the prognosis (**Figure 1B**).

Finally, as shown in Figure, univariate analysis using Cox regression showed that IGF-1 (HR =2.30; P=0.007)were significantly associated with overall survival, IGF-1R (HR =1.00, P=0.004), RAGE (HR =1.10, P<0.001) was not associated with overall survival (**Figure 1C**).

### Basic patient characteristics

A total of 148 patients [61.5 ± 12.9 years; median age: 65 years (interquartile range [IQR]: 52-72 years; 84 men)] were included in this study. There were 106 patients in the CAgroup, including 75 patients with CRC and 31 patients with CRC+T2DM; the control group comprised 42 patients with T2DM. All CRC patients were diagnosed by pathological examination of a surgically excised sample; 56.6% of eslions were ulcerated, and 35.8% were augmented. We performed TNM staging on all patients with CRC, of which 42.5% (45/106) were stage IIIA, 45.2% (48/106) were stage IIIB and 12.3% (13/106) were stage IIIC. Tumour lesions were located in the right hemicocele, transverse colon and descending colon in 72.6% (77/106) and in the sigmoid colon and rectum in 27.4% (29/106). The maximum diameter of the tumour lesions ranged from 1-13.5 cm, with 35.8% (38/106) having a maximum diameter of ≥5 cm (**Table 1**).

**Table 1:** Basic characteristics of the included participants.

CRC+T2DM (n=31)	CRC (n=75)	T2DM (n=42)	
Sex			
Male	48.4% (15/31)	57.3% (43/75)	61.9% (26/42)
Female	51.4% (16/31)	42.7% (32/75)	38.1% (16/42)
Age	68.30 ± 9.79	63.84 ± 11.32	62.35 ± 12.71
BMI	23.80 ± 2.86	23.12 ± 3.82	26.34 ± 4.26
Fasting plasma glucose (mmol/L)	7.18 ± 2.35	5.09 ± 0.94	20.44 ± 9.26
CEA (ng/ml)	5.69(2.85-19.37)	6.55(3.34-15.12)	–
Tumour size(cm)	5.05 ± 2.40	4.49 ± 1.68	–
<5 cm	61.3% (19/31)	64.0% (48/75)	
≥5 cm	38.7% (12/31)	36.0% (27/75)	
Tumour site			–
Colon	38.7% (12/31)	22.7% (17/75)	
Sigmoid colon and rectum	61.3% (19/31)	77.3% (58/75)	
Tumour differentiation -			
Poor	25.8% (8/31)	18.7% (14/75)	
Moderate	54.8% (17/31)	62.6% (47/75)	
Well	19.4% (6/31)	18.7% (14/75)	
Clinical staging			–
IIIA	51.6% (16/31)	40.0% (30/75)	
IIIB	41.9% (13/31)	45.3% (34/75)	
IIIC	6.5% (2/31)	14.7% (11/75)	
Lymphatic metastasis -			
Negative	71.0% (22/31)	52.0% (39/75)	
Positive	29.0% (9/31)	48.0% (36/75)	

### Comparison of serum indicators between the CA and control groups

**Figure 2:**
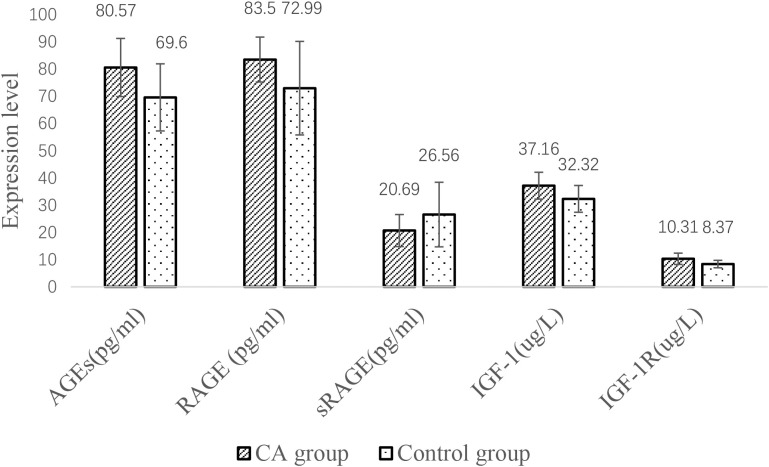
Comparison of serum indicators between CA and control groups.

The serum AGE, RAGE, IGF-1 and IGF-1R levels were higher in the CA group than in the control group, but the sRAGE concentration was lower than that in the control group (P< all 0.05) (**Figure 2**).

### Comparison of the CRC group and CRC+T2DM group

**Figure 3:**
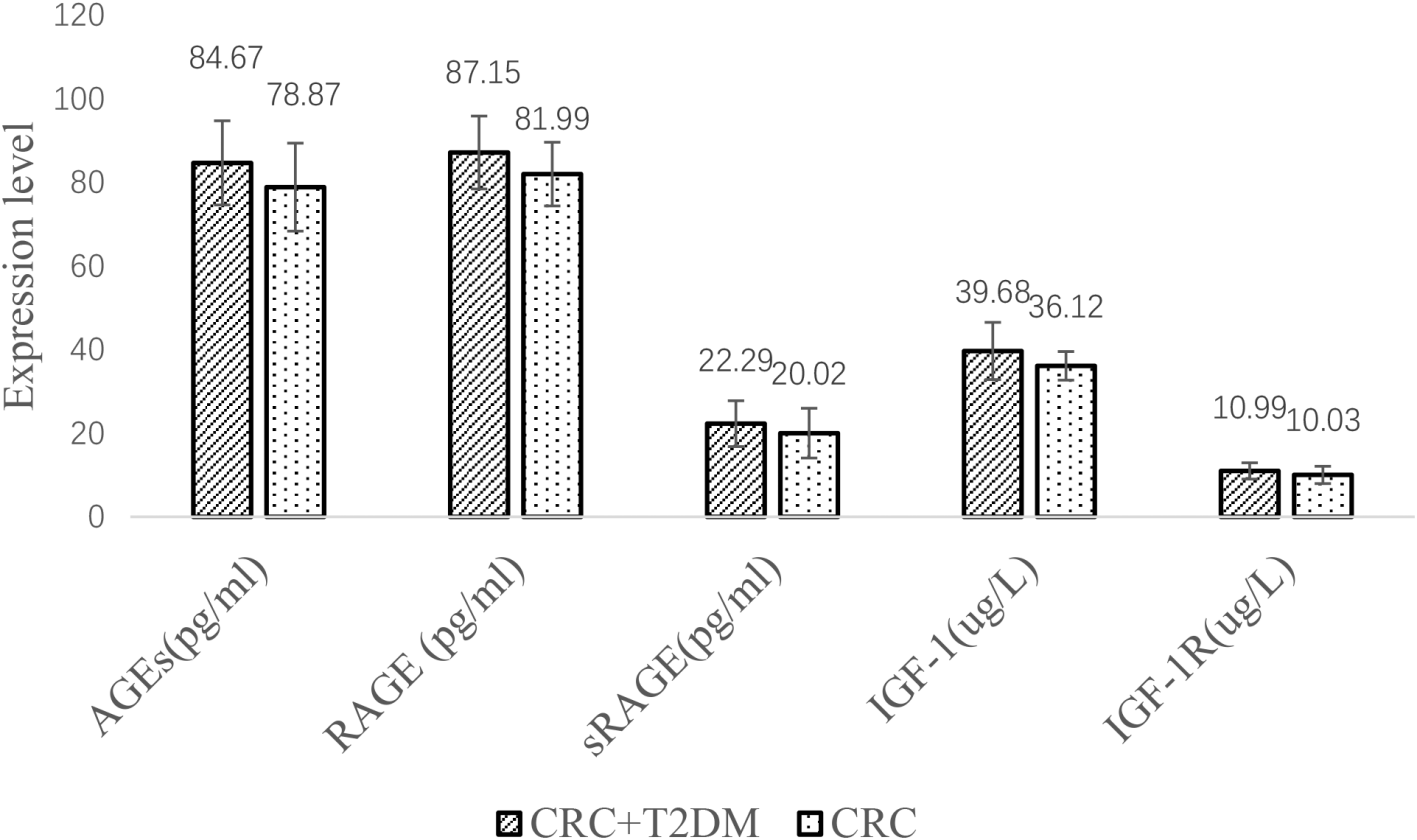
Comparison of the CRC group and CRC+T2DM group.

Serum AGE, RAGE, sRAGE, IGF1 and IGF1R levels were significantly higher in the CRC+T2DM group than in the CRC group (all p < 0.05) (**Figure 3**). Serum sRAGE concentrations were slightly higher in the CRC+T2DM group than in the CRC group, but the differences were not statistically significant (p = 0.064).

### Comparison of serum indicators and different clinical characteristics in CRC+T2DM patients

Serum AGE levels were significantly higher in CRC+T2DM patients ≥55 years old than in patients <55 years old (85.70 ± 9.3pg/ml vs. 69.67 ± 15.12pg/ml), with a statistically significant difference between the two groups (P=0.027). We did not find differences in serum IGF-1, IGF-1R, AGE, RAGE or sRAGE levels in other clinical characteristics of CRC+ T2DM patients (all P > 0.05) (**Table 2**).

**Table 2:** Comparison of the serum indicators of CRC+T2DM patients with different clinical characteristics.

Group	No. of studies	AGEs (pg/ml)	RAGE (pg/ml)	sRAGE (pg/ml)	IGF-1 (µg/L)	IGF-1R (µg/L)
Sex						
Male	15	81.32 ± 11.41	84.37 ± 11.53	24.20 ± 4.99	38.70 ± 8.73	11.38 ± 2.20
Female	16	87.82 ± 7.72	89.76 ± 3.63	20.51 ± 5.46	40.60 ± 4.54	10.63 ± 1.68
Age(years)						
<55	2	69.67 ± 15.12*	83.63 ± 1.96	21.33 ± 7.67	35.49 ± 6.51	11.79 ± 2.77
≥55	29	85.70 ± 9.3	87.40 ± 8.97	22.34 ± 5.48	39.97 ± 6.88	10.93 ± 1.94
Tumour site						
Colon	19	83.53 ± 10.71	87.29 ± 8.16	22.63 ± 5.59	38.83 ± 6.44	11.01 ± 2.03
Sigmoid colon and rectum	12	86.47 ± 9.12	86.93 ± 9.93	21.77 ± 5.51	41.03 ± 7.51	10.96 ± 1.92
Tumour size (cm)						
<5	19	84.94 ± 11.61**	85.42 ± 8.46	22.70 ± 5.67	38.10 ± 7.40	11.17 ± 1.80
≥5	12	94.04 ± 5.03	91.08 ± 7.13	22.06 ± 5.75	39.12 ± 4.69	10.77 ± 2.14
Tumour differentiation						
Poor	8	85.61 ± 11.86	87.01 ± 11.53	21.76 ± 6.45	39.80 ± 8.46	10.78 ± 2.03
Well/moderate	23	84.35 ± 9.65	87.21 ± 7.84	22.48 ± 5.25	39.64 ± 6.41	11.06 ± 1.97
Lymphatic metastasis						
Negative	22	84.46 ± 10.65	88.53 ± 9.24	21.62 ± 4.99	40.47 ± 7.74	10.99 ± 2.03
Positive	9	85.19 ± 9.06	83.79 ± 6.57	23.94 ± 6.56	37.75 ± 3.54	10.99 ± 1.86
BMI (kg/m2)						
<25	20	84.57 ± 8.16	88.67 ± 8.82	21.94 ± 5.96	38.95 ± 7.02	10.51 ± 1.89
≥25	11	84.86 ± 13.32	84.40 ± 8.22	22.94 ± 4.67	41.00 ± 6.62	11.86 ± 1.84
CEA(ng/ml)						
<20	23	84.59 ± 10.75	87.11 ± 9.18	21.76 ± 5.59	39.87 ± 7.57	11.15 ± 2.01
≥20	8	84.89 ± 8.45	87.27 ± 7.81	23.85 ± 5.15	39.14 ± 4.49	10.52 ± 1.82

No., number;BMI, body mass index;CEA, carcinoma embryonic antigen; *P=0.027, **p=0.004.

### Correlation analysis among the serum indicators

**Figure 4:**
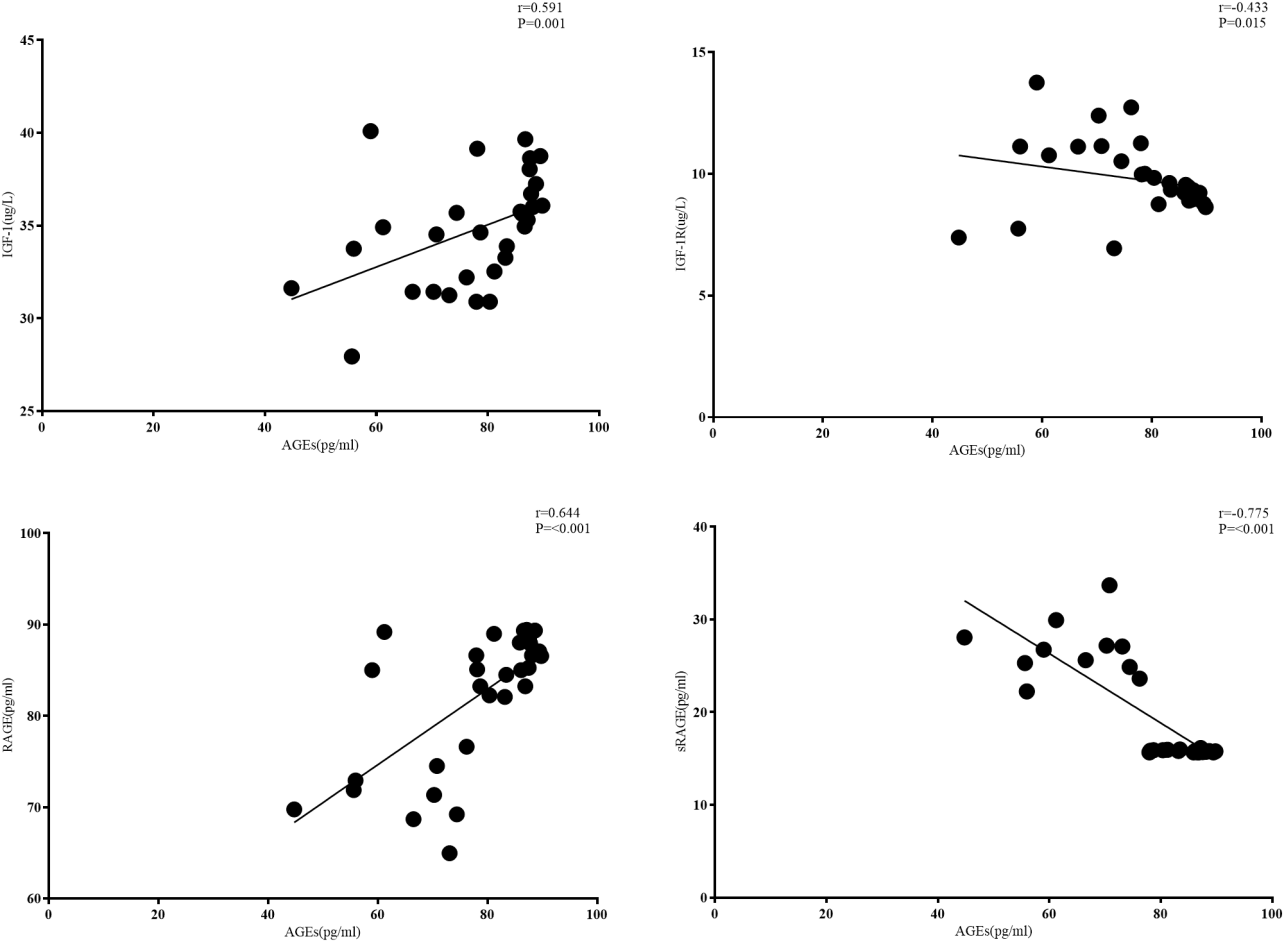
Correlation analysis between serum indices.

As shown in **Figure 1**, for patients with CRC combined with T2DM, serum AGE levels were positively correlated with RAGE levels (r= 0.644, p<0.001) and negatively correlated with sRAGE levels (r= -0.775, p<0.001). Serum AGE levels were positively correlated with IGF-1 levels (r= 0.591, p<0.001) and negatively correlated with IGF-1R levels (r= -0.433, p=0.015). Serum IGF-1 and IGF-1R levels were not correlated with each other (r=-0.12, p> 0.05) (**Figure 4**).

### Logistic regression analysis

**Figure 5:**
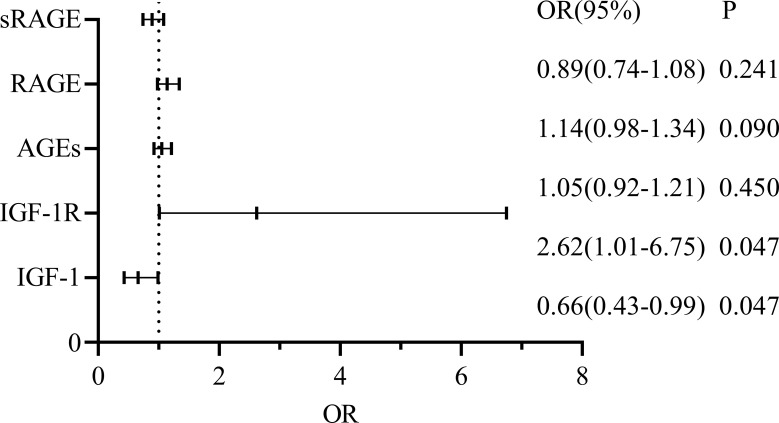
Logistic regression analysis for the CRC+T2DM group.

For CRC+ T2DM patients, sex, age, AGEs, RAGE, sRAGE, IGF1 and IGF1R were included to construct a multifactorial logistic regression analysis, and the results revealed that the effects of age, serum IGF-1 and IGF-1R on the occurrence of CRC in T2DM patients were statistically significant (all p < 0.05), while sex, serum AGEs and RAGE had no statistically significant effect on the development of CRC in patients with T2DM (all p > 0.05) (**Figure 5**).

## Discussion

Studies involving colorectal cancer have revealed that T2DM-related traits, such as hyperglycaemia and hypoinsulinaemia, increase the risk of disease and cancer-specific mortality while lowering disease-free and overall survival in CRC patients (17, 18). Therefore, it was proposed that the dysregulation of the IGF axis, late glycosylation products, hyperglycaemia and hypoinsulinaemia may aid in the development of CRC. Therefore, the current study analysed the relationship between these markers and determined whether serum AGEs, RAGE, sRAGE, IGF-1, and IGF-1R function as influencing variables for the risk of developing CRC in patients with T2DM.

Based on TCGA database analysis, We discovered that the expressions of IGF-1, IGF1R and RAGE in colorectal cancer tissues were different from those in normal tissues. Moreover, To evaluate the prognostic value of CILP2 on the overall survival of CRC patients in TCGA cohort, the Kaplan-Meier and Cox regression analyses were performed. we found that the high expression of IGF-1R and RAGE was significantly correlated with a much poorer prognosis in CRC, and IGF-1R serve as a independent prognostic factors for CRC.

Then, we used experiments to further explore the correlation between IGF-1, AGEs and their receptors and CRC with T2DM.According to our findings, for CRC patients, serum sRAGE levels were lower than in the T2DM group, while circulating AGEs and RAGE levels were higher than in the T2DM group. This study also demonstrated that patients with CRC+T2DM had higher serum levels of AGEs, RAGE, and sRAGE than patients with CRC alone. The activation of RAGE is the primary mechanism linking T2DM and AGEs, and it is currently commonly believed that AGEs are responsible for difficulties associated with T2DM (19). RAGE is an immunoglobulin superfamily member and a cell surface receptor with three extracellular structural domains, a transmembrane structural domain, and a cytoplasmic tail (20). In AGE-RAGE interactions, different signalling pathways can be activated, including the activator protein 1 (AP-1), NF-κB, signal transducer and activator of transcription 3 (STAT3), SMAD family member 4 (Smad4), MAPK (mitogen-activated protein kinase), mammalian target of rapamycin (mTOR), and phosphatidylinositol 3-kinase (PI3K) pathways (21). Through these signalling pathways, CRC onset, development and progression can be promoted (22). First, Kuniyasu et al. (23) demonstrated that RAGE is expressed in every CRC cell line examined and that RAGE cooperates with the epithelial-to-mesenchymal transition pathway to promote the development of cancer stem cells. Furthermore, Deng (24) and colleagues showed that glucose-derived AGEs are elevated in the serum of CRC+ T2DM patients to activate the RAGE/ERK/SP1/matrix metallopeptidase-2 (MMP2) cascade reaction in cancer tissues to promote CRC invasion and metastasis. These AGEs can also activate the RAGE/ERK/SP1/matrix metallopeptidase-2 (MMP2) cascade in cancer tissues.

In our investigation, CRC+ T2DM patients over the age of 55 years had significantly higher serum levels of AGEs than those under the age of 55 years. AGEs might be not only a biomarker but also a potential driver of ageing because they are associated with changes in the ageing process and the emergence of many age-related diseases (25). And CRC is also closely related to age. According to the trend analysis of incidence and mortality of colorectal cancer in China, for age-specific rates, the incidence begins to increase significantly at 40-45 age group and reached a peak at 75; the mortality increased significantly at 45-50. The age effect increased with age in general (26). In combination with our study, there may also be a correlation between high expression of AGEs and increased mortality with age in CRC. Additionally, serum AGEs were higher in these patients (p=0.004), and the tumour lesion diameter was larger. Rahimia et al. (27) also supported the finding that the size of an ovarian cancer tumour was positively correlated with the expression of AGEs and was strongly related to patient prognosis. The likelihood of long-term survival is, in general, higher with a smaller tumour size. Our investigation found no correlation between serum AGE levels and the location of CRC, level of differentiation, presence of lymph node metastases, body mass index, or CEA levels. It has been hypothesized that genetic and environmental factors have a significant impact on the variability of AGE concentrations (28). High AGE expression was discovered in 98.39% (122/124) of colorectal cancer patients in a study by Sacorario et al. (7), and it was slightly higher in patients with mucinous adenocarcinoma (p=0.0997). They further concluded that there was a correlation between the AGE tissue score and the histological tumour grade (Mann Whitney U test I vs. II vs. III, p=0.0157), with lower levels being seen in grade II tumours. The tumour stage and site did not correlate with AGE or RAGE (p>0.10). A predictive role for AGEs in both early and late stages of colorectal cancer may be possible if AGE expression levels correlate with pathological grade [7].

In this investigation, we also discovered that, despite sRAGE levels were lower in the CRC and CRC+T2DM groups than in the T2DM group, respectively, the CRC+T2DM group was still higher than the CRC group. sRAGE has the ability to bind AGEs and serve as a RAGE decoy (28). However,sRAGE binding to AGEs cannot trigger a sig-naling cascade because it has no transmembrane and intracellular domains. For this reason, sRAGE is thought to play a beneficial role by acting as an in-valid receptor that attenuates RAGE-AGEs interac-tions at the cell surface. In diabetes,the circulating sRAGE concentrations are too low to capture and eliminate AGEs produced by hyperglycemia (29). Accumulat-ing evidence has shown that increased levels of circu-lating sRAGE can as one potential marker for the expression of RAGE and activation of the RAGE axis. In addition,according to previous studies, the combination of AGEs with sRAGE did not result in oxidative stress or inflammation, in contrast to RAGE (12). Higher levels of sRAGE are linked to inflammation and an increased risk of several chronic diseases, including cancer (8), despite the possibility that their concentration may not be sufficient to bind all circulating AGEs. The asynchronous variant rs2070600 (Gly82Ser), which is linked to both cancer risk and changes in circulating sRAGE, was used as a surrogate marker in a 2016 Mendelian randomized meta-analysis study [9] to infer a potential causal relationship, suggesting that lowered circulating sRAGE concentrations through genetic mechanisms may increase cancer risk. Additionally, it has been hypothesized that carriers with lower levels of circulating sRAGE are more likely to develop breast, lung, and liver cancer (30–32). We hypothesize that high circulating sRAGE levels are not only a risk factor for patients with T2DM but also a protective factor for patients with CRC based on our research findings and prior literature.

Another biomarker linked to T2DM is IGF-1, which also encourages the growth of CRC. In our investigation, we discovered that circulating IGF-1 and IGF-1R levels were higher in CRC patients than in the T2DM population and lower than in the CRC+T2DM group. The two primary IGF-1 pathways are the PI3K/Akt pathway and the MAPK pathway, both of which are essential for promoting the proliferation of tumour cells (15). Insulin resistance may increase serum insulin levels in T2DM and, by blocking IGF-binding proteins, indirectly increase IGF-1 biological activity (33). Insulin resistance may also increase mitogenic activity. It is accurate to say that increased levels of insulin, IGF-1, and IGF-2 in the blood circulation are directly linked to the onset and spread of the majority of tumours (34). High IGF-1R expression may play a role in tumour progression in CRC, as it is associated with both cell proliferation and differentiation (35), in agreement with our findings.

In the CRC+T2DM group, serum AGEs were found to be negatively connected with sRAGE (r= -0.377, p=0.037) and favourably correlated with RAGE (r= 0.644, p=0.001). According to DENG, R et al. (36), who discovered that AGEs can enhance RAGE expression in a dose-dependent manner in CRC, the positive connection between AGEs and RAGE demonstrates that the two interact with one another to promote the development of CRC. A negative correlation between AGEs and sRAGE suggests that lower sRAGE levels are linked to higher levels of circulating AGEs. A prior study in T2DM showed that changes in plasma sRAGE and AGEs have a weak correlation, indicating that sRAGE serves as a trap for AGEs (37). The correlation in our study was increased when T2DM patients concurrently developed CRC, indicating that sRAGE may be depleted with illness and concurrent disease progression and no longer have the capacity to trap additional AGEs. Additionally, this result supports the hypothesis that circulating sRAGE levels may serve as a protective factor for CRC patients. Previous research has demonstrated that activation of the PI3-kinase-Akt pathway in adipocyte AGEs requires trans-activation of the IGF-1 receptor, a process that involves RAGE, among others. IGF-1 receptors can also be transactivated by RAGE, a member of the immunoglobulin-like superfamily of receptors (15). In our study, we also found that in CRC+ T2DM patients, serum AGEs were positively correlated with IGF-1 levels (r= 0.591, p<0.001) and negatively correlated with IGF-1R levels (r= -0.433, p=0.015), demonstrating that there may indeed be a correlation pathway between AGEs and IGF-1 and IGF-1R promoting an increased risk of CRC in T2DM patients.

Finally, we conducted a logistical multifactorial regression analysis in the CRC+T2DM group and found that age, IGF-1, and IGF-1R were significant predictors of CRC development in T2DM patients (p<0.05) after adjusting for age and sex as confounders or potential confounders. Previous research has shown that age does play a significant role in the development of CRC and that the risk of CRC increase with age (38). While older patients have lower overall survival rates than younger patients, it is noteworthy that at the age of 80 years, age ceases to be a significant risk factor for CRC (39). We discovered through regression analysis that IGF-1 and IGF-1R had independent impacts on the development of CRC in T2DM, whereas AGEs, RAGE, and sRAGE did not. Further evidence that IGF-1 can encourage the development of CRC in T2DM patients was found to be provided by the association between each unit increase in IGF-1R and an approximately 0.96-fold increase in the probability of developing CRC. Based on the results of our confirmatory analysis and previous studies, we found that IGF-1 overexpression was previously thought to be a risk factor for CRC (40), but we found that IGF-1(r=2.3,P=0.007) is actually a protective factor for the development of CRC in the group of patients with type 2 diabetes. According to Rui Liu et al. (40), variations in IGF-1 levels are not directly related to the characteristics of the malignant cells themselves but rather to the aggressiveness and expansion of CRC and its subsequent systemic effects. The TNM staging of our sample, on the other hand, was predominantly T3 and quite single, which could have had an impact on our analysis of how IGF-1 affects CRC. We hypothesized that AGEs do not directly cause CRC but rather indirectly affect CRC development by acting on IGF-1 and its receptors in our previous comparison of the CRC+T2DM and CRC groups. We found differences in the circulating levels of serum AGEs and correlations with both IGF-1 and IGF-1R in the CRC+T2DM group. Some investigators have also found that diabetic complications and other diseases associated with AGEs may be treated with IGF-1 receptor antagonists and cholesterol-depleting agents (8). In addition, there was no correlation between IGF-1 and its receptor levels in this study, which may be related to the limited sample size, and their relationship needs to be further investigated.

IGF-1 receptor antagonists and cholesterol-lowering medications may be used to treat AGE-related disorders such as diabetic complications, according to some researchers (8). Further research is required because there was no link between IGF-1 and its receptor levels in this study, which may have been due to uneven staging sample

In conclusion, our investigation discovered that circulating levels of IGF-1, IGF-1R, AGEs, and RAGE were high in CRC patients, suggesting a potential link between T2DM and CRC development. With regard to patient age and tumour size, AGEs were positively correlated with RAGE and negatively correlated with sRAGE. Additionally, we discovered that in patients with T2DM, IGF-1 and IGF-1R were independent factors promoting the development of CRC. Furthermore, IGF-1 and IGF-1R were correlated with AGEs in CRC patients who also had T2DM, suggesting that AGEs may influence the development of CRC in T2DM patients. These findings suggest that we may be able to lower the risk of CRC in the clinic by regulating AGEs through the regulation of blood glucose levels, which will affect IGF-1 and its receptors. We will delve deeper into the particular mechanisms and analyse a larger sample in depth in future studies.

## Data availability statement

The raw data supporting the conclusions of this article will be made available by the authors, without undue reservation. Requests to access the datasets should be directed to 316204349@qq.com.

## Ethics statement

This study has been reviewed and approved by Harbin Medical University, KY2020-022. The written informed consent of all participants in this study has been obtained.

## Author contributions

All authors contributed to the study conception and design. Material preparation, data collection and analysis were performed by CZ. The first draft of the manuscript was written by CZ and all authors commented on previous versions of the manuscript. All authors read and approved the final manuscript.
